# Tilapia Piscidin 4 (TP4) Stimulates Cell Proliferation and Wound Closure in MRSA-Infected Wounds in Mice

**DOI:** 10.3390/md13052813

**Published:** 2015-05-06

**Authors:** Hang-Ning Huang, Yi-Lin Chan, Chang-Jer Wu, Jyh-Yih Chen

**Affiliations:** 1 Marine Research Station, Institute of Cellular and Organismic Biology, Academia Sinica, 23-10 Dahuen Road, Jiaushi, Ilan 262, Taiwan; E-Mail: henryhw910@yahoo.com.tw; 2 Department of Life Science, Chinese Culture University, Taipei, Taiwan; E-Mail: phd.elainechan@gmail.com; 3 Department of Food Science, National Taiwan Ocean University, 2, Pei-Ning Road, Keelung, Taiwan

**Keywords:** antimicrobial peptides, tilapia piscidin 4, wound healing, *Staphylococcus aureus*

## Abstract

Antimicrobial peptides (AMPs) are endogenous antibiotics that directly affect microorganisms, and also have a variety of receptor-mediated functions. One such AMP, Tilapia piscidin 4 (TP4), was isolated from Nile tilapia (*Oreochromis niloticus*); TP4 has antibacterial effects and regulates the innate immune system. The aim of the present study was to characterize the role of TP4 in the regulation of wound closure in mice and proliferation of a keratinocyte cell line (HaCaT) and fibroblast cell line (Hs-68). *In vitro*, TP4 stimulated cell proliferation and activated collagen I, collagen III, and keratinocyte growth factor (KGF) gene expression in Hs-68 cells, which induces keratin production by HaCaT cells. This effect was detectable at TP4 concentrations of 6.25 µg/mL in both cell lines. *In vivo*, TP4 was found to be highly effective at combating peritonitis and wound infection caused by MRSA in mouse models, without inducing adverse behavioral effects or liver or kidney toxicity. Taken together, our results indicate that TP4 enhances the survival rate of mice infected with the bacterial pathogen MRSA through both antimicrobial and wound closure activities mediated by epidermal growth factor (EGF), transforming growth factor (TGF), and vascular endothelial growth factor (VEGF). The peptide is likely involved in antibacterial processes and regulation of tissue homeostasis in infected wounds in mice. Overall, these results suggest that TP4 may be suitable for development as a novel topical agent for wound dressing.

## 1. Introduction

Antibiotic resistance is recognized as a major problem worldwide in the management of infectious disease, both in hospital settings and in the community. Therefore, there is a clear requirement for new antibiotics, particularly those effective against multidrug-resistant bacteria [1]. Cases of wound infection due to multidrug-resistant organisms, such as methicillin-resistant *Staphylococcus aureus* (MRSA), continue to increase. At the same time, there has been a decline in the development of new antibacterial therapies [2].

Antimicrobial peptides (AMPs) are endogenous antibiotics that directly target microorganisms [3]. In addition to host defense, they are also involved in the modulation of the immune response [4]. Tilapia piscidin 4 (TP4) is an AMP isolated from Nile tilapia (*Oreochromis niloticus*), and was characterized as early as 2012. Tilapia piscidin 4 is a 23 amino acid peptide that starts with phenylalanine (F) and ends with histidine (H) [5]. TP4 is a pore-forming peptide with an α-helix structure, which confers selective cytolytic activity against bacteria. In addition to disrupting bacterial membranes, Tilapia α-helix AMPs have been reported to stimulate immunogenicity, induce a TH1 cellular immune response, and act as adjuvants to vaccines in fish [6]. TP4 has antimicrobial activity against both Gram-positive and -negative bacteria [5]. Furthermore, clinical case studies have shown that application of AMPs to severely infected cutaneous wounds can clear the infection and improve healing [7]. In addition, previous studies have confirmed that AMPs have immunomodulatory function [8]. A recent study reported that AMPs may promote resistance to bacterial infections by stabilizing the cytoskeleton network in host cells [9]. Thus, TP4 has many features consistent with antibiotics, but potentially has broader applications, and may eliminate or reduce concerns of bacterial resistance.

Cutaneous wound healing is a complex process involving blood clotting, inflammation, new tissue formation, and finally, tissue remodeling [10]. The inflammation process is associated with epithelial injury and with increased expression of mediatory molecules. Some of these molecules, such as keratinocyte growth factor [11] or epidermal growth factor (EGF) [12,13], are involved in the regulation of epithelial repair processes. AMPs also regulate epithelial reconstitution, while human neutrophil defensins induce airway epithelial proliferation and wound closure [14,15].

Treatment with effective AMPs can both help reduce the risk of infection, and reduce the overall time required for wound healing. Bacteria can colonize wounds within 48 h of injury, and bacteria such as *Staphylococcus aureus*, *Pseudomonas aeruginosa*, and *Streptococcus* spp. may prolong the inflammatory phase of wound healing [16]. Topical or systematic application of suitable antimicrobial agents may prevent wound infection and/or accelerate wound healing. Inflammation involves the release of biologically-active mediators, which attract macrophages and lymphocytes; these cells secrete cytokines that stimulate epithelial proliferation, and result in the chemoattraction of epithelial cells to the wound area [17].

The goal of the current study was to examine the antimicrobial, anti-inflammatory, and wound healing properties of TP4 treatment in MRSA-infected mice. We investigated whether treatment of a mouse model with peptide (AMPs) can (i) enable the identification of novel candidates for antibacterial therapeutic drugs; (ii) inhibit bacterial growth; and (iii) accelerate wound closure.

## 2. Results

### 2.1. In Vitro Toxicity and Stimulation of Proliferation by TP4

**Figure 1:**
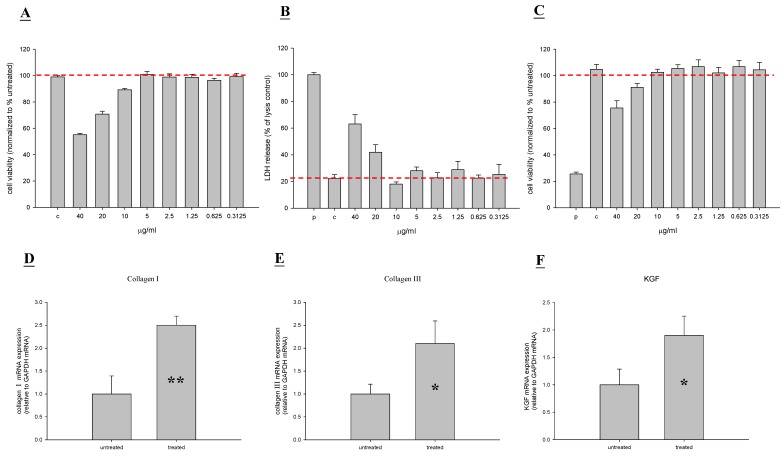
Tilapia piscidin 4 (TP4) does not exhibit cytotoxic effects on a human fibroblast cell line (Hs68), and actually stimulates proliferation activity. (**A**–**C**) Hs68 cells were treated with different doses of TP4 for 48 h, and cell viability was measured by neutral red, LDH, and MTT assays; (**D**–**F**) Experimental Hs68 cells were treated with TP4 (6.25 μg/mL), while control cells were untreated. After 48 h, total RNA was isolated and reverse transcribed for use in real-time qPCR analysis. Values with different letters show significant differences (*P* < 0.05), as determined by ANOVA. The lowercase letters indicate the following: n.s., not significant; * significant (*P* < 0.05); ** significant (*P* < 0.001).

We first studied the cell toxicity of TP4 in a fibroblast cell line (Hs-68) and keratinocyte cell line (HaCaT) using neutral red, LDH, and MTT assays; we observed that TP4 at various concentrations up to 20 μg/mL affects cell viability in the Hs-68 line (Figure 1A–C). In addition, cell proliferation was significantly increased by low doses (2.5~10 μg/mL), as reflected by the change in cell viability as compared to the untreated group. We subsequently investigated the effect of TP4 on cell proliferation factors. Collagen and keratinocyte growth factor (KGF) are important performance factors for cell proliferation [18]. We thus analyzed collagen I and III, and KGF gene expression in TP4 treated Hs-68 cells. Expression of all genes was enhanced by TP4 treatment, as compared to expression in the controls (Figure 1D–F).

**Figure 2:**
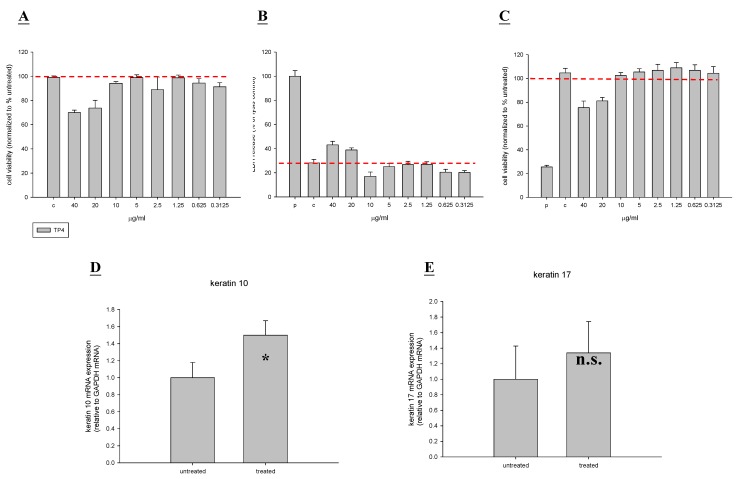
Tilapia piscidin 4 (TP4) does not exhibit cytotoxic effects on a human keratinocyte cell line (HaCaT), and actually stimulates proliferation activity. (**A**–**C**) HaCaT cells were treated with different doses of TP4 for 48 h, and cell viability was measured by neutral red, LDH, and MTT assays; (**D**–**E**) Experimental HaCaT cells were treated with TP4 (6.25 µg/mL), while control cells were untreated. After 48 h, total RNA was isolated and reverse transcribed for use in real-time qPCR analysis. Values with different letters show significant differences (*P* < 0.05), as determined by ANOVA. The lowercase letters indicate the following: n.s., not significant; * significant (*P* < 0.05); ** significant (*P* < 0.001).

TP4 at tested concentrations (down to 10 μg/mL) did not affect the viability of HaCaT cells (Figure 2A–C). Of the proliferation and differentiation mediators in this keratinocyte cell line (HaCaT), keratin 10 and 17 are of particular importance, because they play a major role in coordinating protein synthesis and cell growth mechanisms [19]. As shown in Figure 2D, TP4 significantly increased gene expression of keratin 10, but did not affect that of keratin 17 (Figure 2E). Therefore, our results indicate that TP4 enhances Hs-68 and HaCaT cell proliferation, possibly through activation of the genes encoding collagen I and III, KGF, and keratin 10.

### 2.2. Acute Toxic Effects of TP4 in Mice

We examined the toxicity of TP4 by delivering it via intramuscular (i.m.) injection into mice, and subsequently measuring biochemical factors in the blood. Mice treated with 2 mg of TP4 did not exhibit any significant changes in the levels of blood urea nitrogen (BUN), creatinine (CRE), total glucose (GLU), or creatine phosphokinase (CPK) (Table 1). While glutamic oxaloacetic transaminase (GOT) and glutamic pyruvic transaminase (GPT) were significantly increased at the first day after injection, they eventually returned to normal levels (Table 1). Our results suggest that TP4 does not induce systemic toxic effects, even at the highest concentration tested (2 mg/mouse).

**Table 1:** Biochemical parameters of mice after intramuscular injection of TP4 (2 mg/mouse). The following parameters were measured in the blood: glutamic oxaloacetic transaminase (GOT), glutamic pyruvic transaminase (GPT), creatinine (CRE), blood urea nitrogen (BUN), and creatine phosphokinase (CPK). Values with different letters show significant differences (*P* < 0.05) between time points, as determined by ANOVA.

	Control (*n* = 10)			TP4 (*n* = 10)		
Time (Day)	1	3	6	1	3	6
**GOT (U/L)**	42.1 ± 1.3 ^A^	44.2 ± 2.1 ^A^	41.6 ± 1.5 ^A^	102.1 ± 32.1 ^C^	43.2 ± 1.5 ^A^	45 ± 6.1 ^A^
**GPT (U/L)**	45.2 ± 1.1 ^A^	43.4 ± 4.3 ^A^	46.1 ± 3.3 ^A^	81.2 ± 5.2 ^C^	41.2 ± 4.7 ^A^	48 ± 3.7 ^A^
**CRE (mg/dL)**	0.2 ± 0.1 ^A^	0.6 ± 0.3 ^A^	0.4 ± 0.2 ^A^	0.3 ± 0.1 ^A^	0.5 ± 0.1 ^A^	0.5 ± 0.1 ^A^
**BUN (mg/dL)**	13.1 ± 2.1 ^A^	14 ± 0.3 ^AB^	16.1 ± 1 ^A^	14.2 ± 1.4 ^A^	15.4 ± 1.3 ^A^	16.2 ± 1 ^A^
**GLU (mg/dL)**	206.0 ± 11.1 ^A^	213.1 ± 11.3 ^A^	208 ± 13.1 ^A^	238.6 ± 32.1 ^AB^	209.1 ± 7.2 ^A^	220.1 ± 23.5 ^A^
**CPK (U/L)**	101.3 ± 1.7 ^A^	110.1 ±6.3 ^A^	104.9 ± 4.2 ^A^	109 ± 11.1 ^A^	108.1 ± 3.2 ^A^	109 ± 25.3 ^AB^

All data are expressed as means ± SD and were compared with the ANOVA (*n* = 10).

### 2.3. TP4 Enhances the Survival of Mice Infected with MRSA

**Figure 3:**
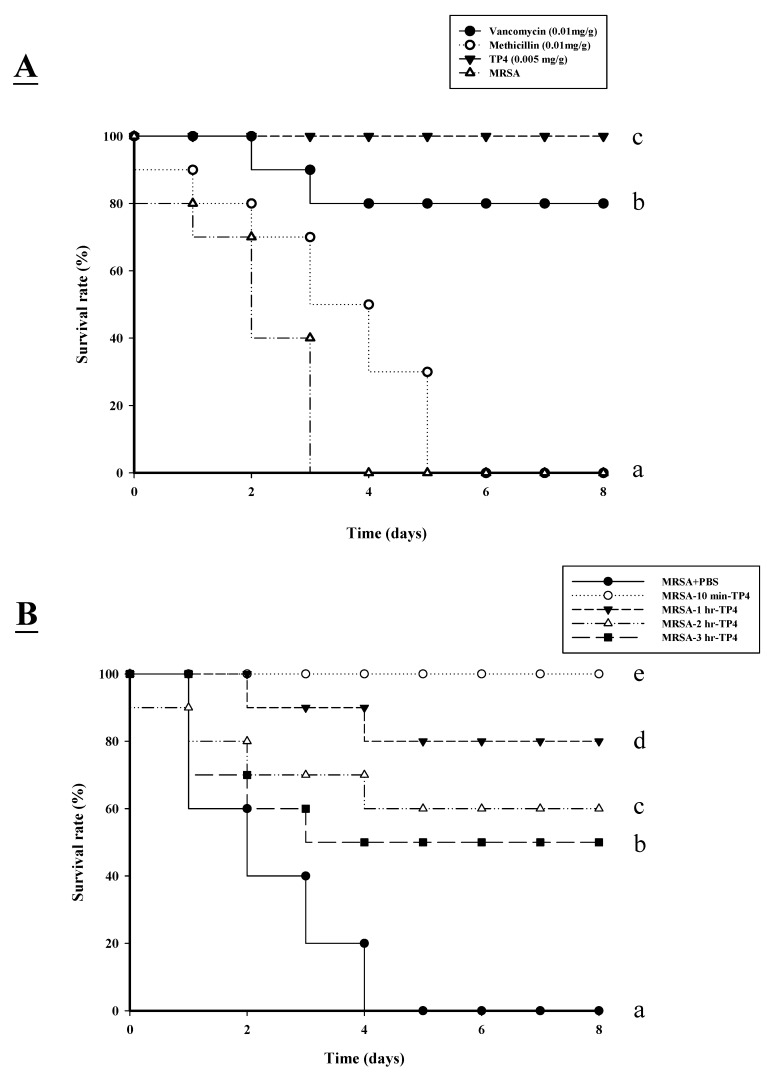
Effects of tilapia piscidin 4 (TP4) treatment on mice infected with MRSA. (**A**) Mice were injected with MRSA (1 × 10^6^ CFU/mouse), and independent groups (*n =* 10) were subsequently injected with TP4, vancomycin, or methicillin. The survival rate was monitored on a daily basis for up to eight days; (**B**) To determine curative potential, mice were first injected with MRSA (1 × 10^6^ CFU/mouse) and then with TP4 (0.005 mg/g) 10, 60, 120, or 180 min later. At these injection times, the MRSA experimental groups exhibited survival rates of 100%, 80%, 60%, and 50%, respectively.

We proceeded to investigate the bactericidal effects of TP4 *in vivo*, by monitoring the survival of mice infected with MRSA prior to treatment with TP4 or antibiotic. All untreated mice infected with MRSA died within 72 h of infection, whereas co-treatment with TP4 decreased the mortality rate (Figure 3A). At eight days after MRSA infection, the survival rates were 100%, 80%, and 0% for mice treated with TP4 (0.005 mg/g), vancomycin (0.01 mg/g), and methicillin (0.01 mg/g), respectively. At 48 h, the rate of lethality in the untreated and infected mice was 60%; treatment with TP4 or vancomycin significantly decreased the rate of mortality (Table 2). Bacteriologic evaluation revealed that untreated mice infected with either strain exhibited 100% positive blood cultures and a high level of bacterial colonization (with the numbers of CFU/mL being no lower than 10^6^) for all organs tested (Table 2). TP4 treatment significantly reduced the bacterial burden in all examined organs compared to those of untreated controls (*P* < 0.05). These data indicate that TP4 can efficiently control MRSA in the organs of infected mice.

To determine the curative potential, mice were first injected with MRSA and then injected with TP4 (0.005 mg/g) 10, 60, 120, or 180 min later. At these injection times, the MRSA experimental groups exhibited survival rates of 100%, 80%, 60%, and 50%, respectively (Figure 3B). The survival rates of mice treated with TP4 were consistently greater than those of untreated mice (PBS-treated control mice). These data indicate that immediate application of TP4 (0.005 mg/g) is important to prevent severe infection. Application within 10–60 min of MRSA infection enabled TP4 to act as an effective curative agent. As such, we next investigated whether TP4 promotes wound repair.

**Table 2:** Bacterial counts at 48 h after the last treatment in the indicated organs of mice infected with MRSA. Infected mice were untreated, or treated with TP4, methicillin, or vancomycin via i.p. injection. Bacterial numbers in blood, peritoneum, spleen, liver, and mesenteric lymph nodes were subsequently recorded. Colony counts from the diluted bacterial solutions are expressed relative to those at the start of treatment. Values with different letters show significant differences (*P* < 0.05) between the group, as determined by ANOVA.

Strain and Treatment	% lethality	Mean ± SD Count (CFU/mL)				
		Blood	Peritoneum	Spleen	Liver	Mesenteric Lymph Nodes
MRSA + PBS	60 ^C^	6 × 10^8^ ± 1.9 × 10^7 C^	2.0 × 10^10^ ± 1.6 × 10^9 C^	5.9 × 10^9^ ± 2.0 × 10^8 C^	2.1 × 10^8^ ± 9.2 × 10^7 C^	3.2 × 10^8^ ± 1.3 × 10^7 C^
MRSA + Methcillin (0.01 mg/g)	20 ^B^	4.1 × 10^7^ ± 1.3 × 10^7 B^	2.3 × 10^9^ ± 2 × 10^8 B^	1.5 × 10^8^ ± 2.1 × 10^7 B^	2.7 × 10^8^ ± 3.3 × 10^6 B^	4.1 × 10^7^ ± 3 × 10^7 C^
MRSA + Vancomycin (0.01 mg/g)	10 ^AB^	8 × 10^4^ ± 1.1 × 10^4 B^	1.5 × 10^6^ ± 1 × 10^5 A^	1.4 × 10^7^ ± 1.1 × 10^6 B^	1.3 × 10^7^ ± 1.8 × 10^5 B^	1 × 10^7^ ± 4 × 10^6 B^
MRSA + TP4	0 ^A^	0 ^A^	1 × 10^6^ ± 1 × 10^6 A^	3 × 10^6^ ± 1.2 × 10^6 A^	2.4 × 10^6^ ± 1 × 10^6 A^	6 × 10^5^ ± 1 × 10^5 A^
(0.005 mg/g)						

Lethality was monitored for 2 day following the injection of TP4 or antibiotic.

### 2.4. Efficacy of TP4 on in Vivo Wound Closure

**Figure 4:**
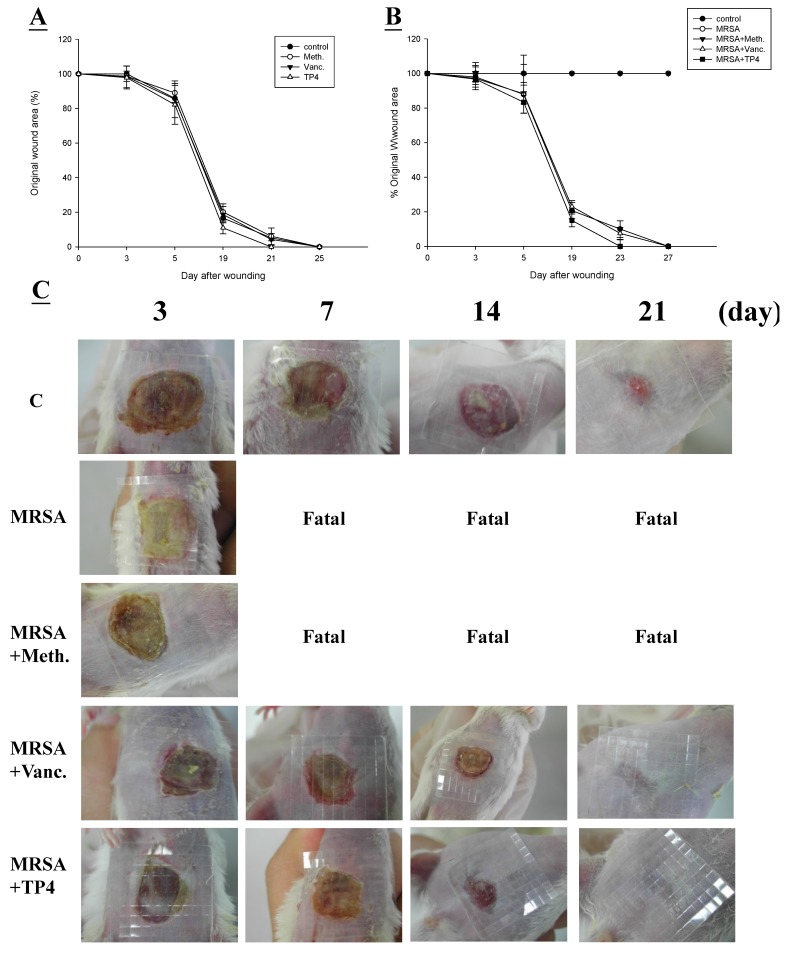
Closure of clean and contaminated wounds. The areas of full-thickness wounds (initially 1 cm in diameter) were measured from the time of wounding until closure. (**A**) All full-thickness aseptic wounds closed by day 25. Meth., methicillin; Vanc., vancomycin; (**B**) Full-thickness wounds contaminated with microorganisms increased in size initially, while TP4-treated wounds did not exhibit the initial expansion and closed somewhat faster (day 21) than vancomycin-treated wounds; (**C**) Photographs of representative wounds. “Fatal” indicates that no mice survived.

First, we examined whether TP4 promotes healing of wounds made in an aseptic manner (Figure 4A). We did not observe any statistical difference between the areas of untreated wounds and Tegaderm™ or antibiotic-treated wounds, with all closing by day >25. This was not unexpected, as skin wounds heal efficiently in healthy mice, and it is unlikely that this process can be significantly improved. Treatment with vancomycin resulted in a similar wound closure time to the control, while wound closure was accelerated by treatment with TP4 alone. Such an increase in wound closure was not observed in uncontaminated wounds, suggesting that TP4 may facilitate wound recovery by combating infection. Unlike the uncontaminated wounds, wound size was largely unchanged after one week in all treatment groups (Figure 4B). By 14 days, wound size in the TP4-treated group was smaller than that of the vancomycin-treated group (*P* < 0.05) (Figure 4C). However, both groups demonstrated full closure by the end of the 27th day.

### 2.5. TP4 Reduced Inflammatory Cytokines

**Figure 5:**
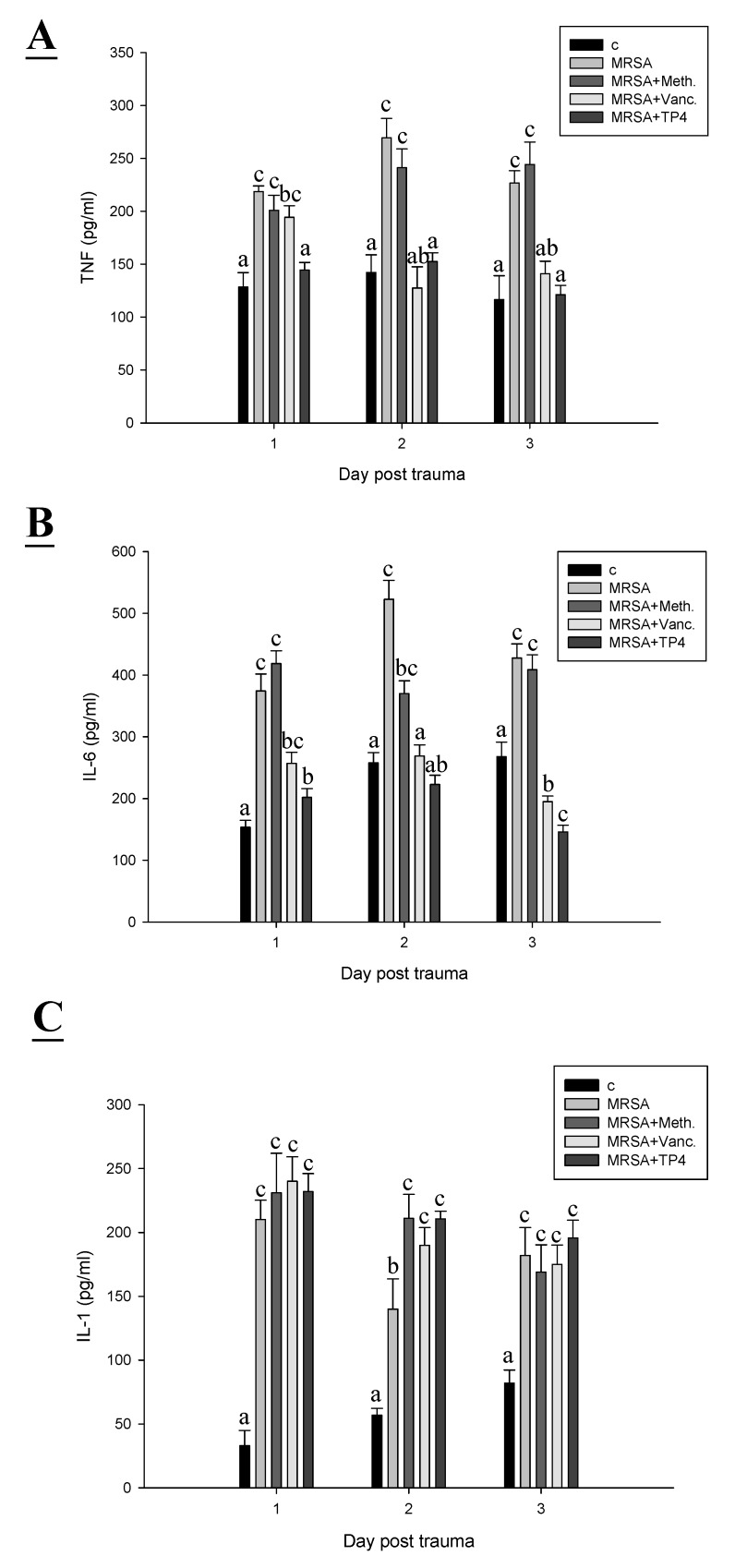
TP4 modulates MRSA mediated-induction of TNF, IL-6, and IL-1. A skin region of about 1 square centimeter was removed from the abdomen of non-anaesthetized mice, and the wound was infected with 50 µL of broth mix containing 10^6^ CFU of MRSA alone, or together with methicillin, vancomycin, or TP4. At different days post-infection, (**A**) TNF; (**B**) IL-6; and (**C**) IL-1 were detected in serum by ELISA. Values with different letters show significant differences (*P* < 0.05) between time points, as determined by ANOVA.

We next examined the direct antimicrobial activity of TP4. The ability of TP4 to modulate cytokines of mice was measured by ELISA (Figure 5A–C). The proinflammatory cytokine IL-6 acts as a potent modulator of innate immunity, while the chemokine monocyte chemoattractant protein 1 (MCP-1) enhances the recruitment of monocytes and macrophages to tissue surrounding wounds [20]. We analyzed serum chemokine and cytokine levels in MRSA-infected mice at three days after treatment. MRSA-infected mice were used as a positive control to confirm cytokine activation. TP4 treatment decreased induction of IL-6 and TNF, compared to expression in the positive controls (Figure 5A,B). In addition, the interleukin-1 (IL-1) protein is important for skin function, which enhances epidermal wound healing [21]. This study shows a statistically significant enhancement of IL-1 in TP4-treated wounds as compared with control wounds (Figure 5C).

### 2.6. TP4 Alters Cell Proliferation Gene Expression Profiles in MRSA-Infected Mice

**Figure 6:**
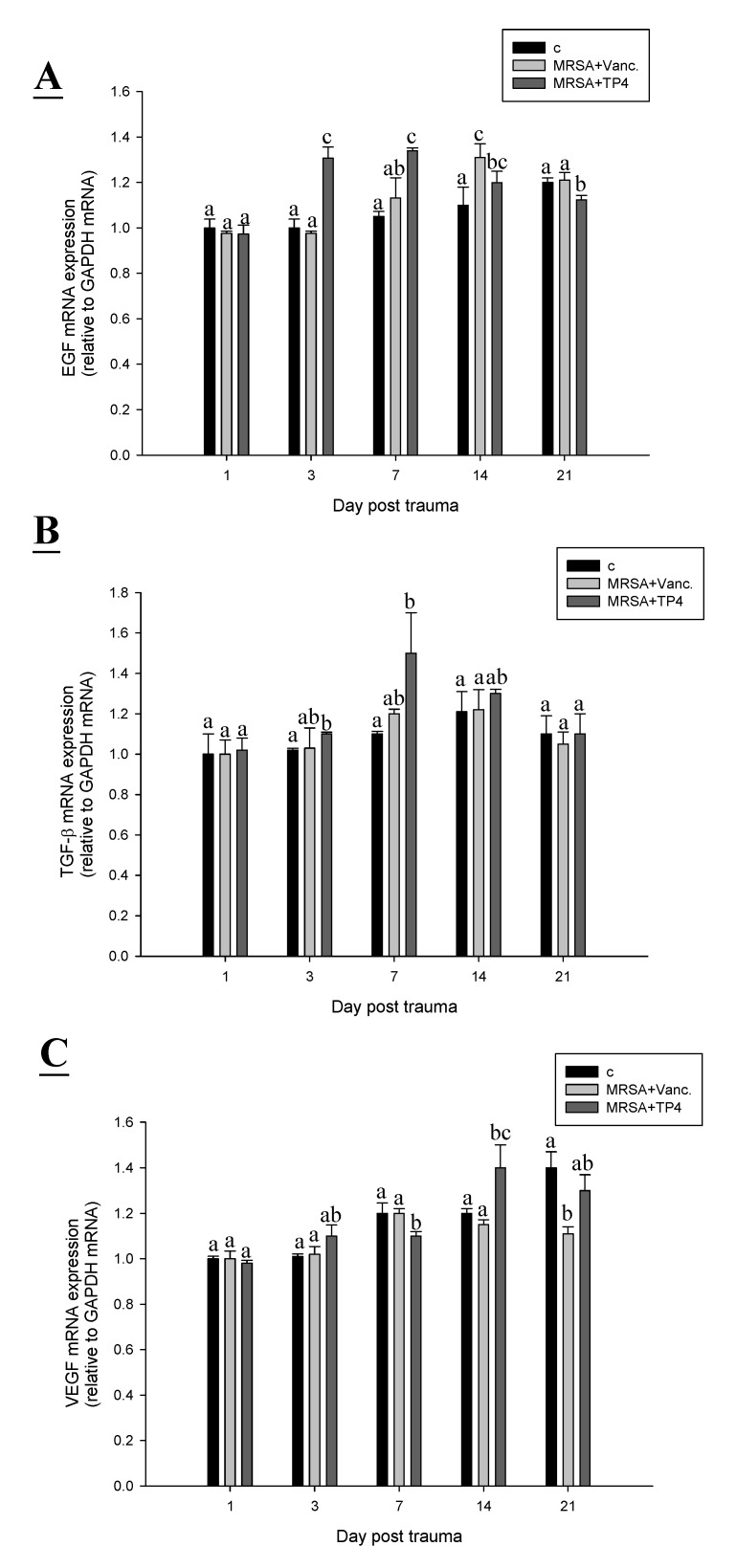
TP4 affects expression profiles of cell proliferation genes in infected wounds of mice. Adult mice were infected with MRSA, and treated with TP4 or antibiotics, while controls were infected but untreated. On the indicated days, total RNA was isolated from the wound and reverse transcribed for use in real-time qPCR analysis of EGF (**A**); TGF-β (**B**); and VEGF (**C**) gene expression. Values with different letters show significant differences (*P* < 0.05) between time points, as determined by ANOVA.

**Figure 7:**
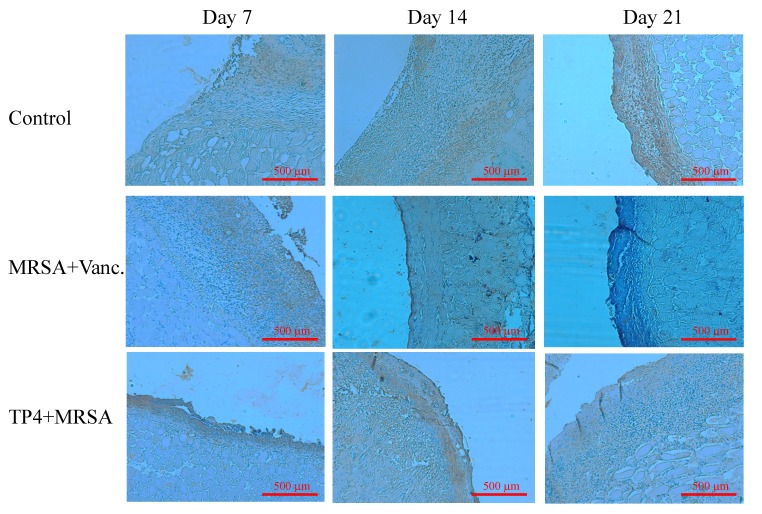
Treatment of infected mice with TP4 enhances VEGF expression. Mice were sacrificed on days 7, 14, or 21 after wounding. Cryosections of wound sites were fixed in formaldehyde, and immunohistochemical analysis was performed using specific antibodies against VEGF.

During wound healing, monocytes begin to replace neutrophils at 48 h, in order to remove wound debris; this is followed by the proliferation phase at 72 h, during which time several growth factors are induced [22,23]. Epidermal growth factor (EGF), transforming growth factor beta (TGF-β), and vascular endothelial growth factor (VEGF) mediate cellular proliferation, regulate differentiation, and stimulate vasculogenesis and angiogenesis, respectively [24]. To examine the expression profiles of cell proliferation genes in MRSA-infected mice treated with TP4, we subjected RNA, extracted from wound tissue of mice on days 1, 3, 7, 14, and 21 post-infection, to real-time RT-PCR. TP4 treatment enhanced gene expression of EGF (3 days), TGF (seven days), and VEGF (14 days) as compared to expression in control and vancomycin-treated mice (Figure 6A,B). Histological examination of biopsies from TP4-treated wounds indicated complete and architecturally normal epidermal regeneration (Figure 7). This study suggests that topical administration of TP4 may be useful for the promotion of wound healing.

## 3. Discussion

A wound agent should address all aspects of healing; it should not only promote tissue regeneration, but also induce hemostasis and limit microbial infection. These latter two processes are critical, as failure to accomplish these immediate and early steps prevents subsequent repair. Here, we have demonstrated that TP4 both stimulates cell proliferation and exerts antibacterial activity.

Antimicrobial peptides are classically regarded as endogenous antibiotics that provide a first line of host defense until other components of the innate immune system or adaptive immune system become active [25]. Increasing evidence from *in vivo* studies derived during the last few years indicates that host defense is indeed one of the main functions of vertebrate AMPs [3]. In parallel, it has become clear that AMPs may possess additional functions. It is known that several AMPs bind to cellular receptors and induce specific cellular reactions [26]. Human neutrophil defensins induce lung epithelial cell proliferation and wound closure *in vitro* [14,15]. Also, TP4 appears to be involved in the regulation of certain processes of epithelial cells.

Here, we observed that TP4 may modulate epidermal repair through control of fibroblast and keratinocyte proliferation and differentiation. We report that the effect of TP4 on keratinocyte cell line (HaCaT) and fibroblast cell line (Hs-68) proliferation may be mediated through activation of collagen I, collagen III, keratinocyte growth factor (KGF), and keratin 10 gene expression. In addition, we used a clinically relevant model (suitable for elucidating the pathophysiology underlying the impairment of wound healing and for testing novel therapeutic agents) to further examine the utility of TP4.

In the current study, TP4 was demonstrated to have anti-bacterial activity *in vitro*, consistent with a previous report that TP4 inhibits bacterial growth [5]. Peptide-based wound healing studies have been reported previously [2], and we have applied this platform to the study of TP4. *In vivo*, TP4 exhibited strong antimicrobial activity, evident within 60 min of exposure. TP4 treatment caused a decrease in TNF and IL-6 at the site of infection on days 1, 2 and 3, as compared with the MRSA infection group. Previously, it was reported that both Gram-negative (LPS) and Gram-positive (lipoteichoic acid) signature molecules caused up-regulation of pro-inflammatory cytokines, through processes that were suppressed by cationic peptides [14]. The cytokine IL-1 plays a major role in innate immune activation during wound healing [27]. Accumulation of macrophages and lymphocytes at the wound-healing site produces inflammatory responses, which are mediated by gene expression of the chemokine CXCL5 [28]. Although TP4 treatment caused a modest increase in IL-6 as compared to the control, this was lower than that induced by MRSA on day 1. The anti-inflammatory effect of TP4 may be due to contributions from several related mechanisms, including that of IL-10 [25]. Furthermore, TP4 reduced MRSA-induced TNF at the wound site on day 1.

Drug development efforts focusing on the regulation of the innate defense system have been limited, in part, because of the potential for inducing harmful sepsis responses [29]. Indeed, most antibiotics stimulate the release of bacterial pathogen-associated signature molecule components [30], and thus contribute to the risk of damaging inflammation and sepsis. We have identified that TP4 can direct kill pathogens and reduce inflammation caused by infection, thereby providing prophylaxis or treatment for a broad spectrum of infections, while balancing or controlling the attendant inflammatory response. Multiple factors, including cytokines, enzymes, and growth factors, are involved in the initiation of the complex process of repair. Recently, IL-1 has been reported to enhance epidermal wound healing [31]. However, whether IL-1 directly regulates wound closure is unknown. Here, we show that IL-1 expression increases over time in MRSA-infected wounds in mice. TP4 was also observed to induce epidermal growth factor (EGF), transforming growth factor (TGF), and vascular endothelial growth factor (VEGF), which may enhance wound closure activity.

## 4. Experimental Section

### 4.1. Bacteria, Cells, and Mice

An MRSA strain commonly associated with human wound infections was selected to generate a polymicrobial solution [32]. The MRSA strain is a clinical isolate from stool obtained from Taipei City Hospital (Heping Fuyou branch). The strain was identified by routine laboratory methods and stored in 20% (vol/vol) glycerol at −80 °C. Tryptic Soy Broth (TSB) was used as the culture medium. HaCaT human keratinocyte cell line and Hs-68 human foreskin fibroblast cell line were grown in DMEM containing 10% (v/v) FBS, 0.37% (w/v) NaHCO_3_, penicillin (100 unit/mL), streptomycin (100 μg/mL), 0.1 mM NEAA, and 1 mM sodium pyruvate at 37 °C in a humidified incubator under 5% CO_2_ and 95% air. The cells were harvested at *ca.* 90% confluence (*ca.* 10^6^ cells/10-cm dish). Balb/c female mice were used for the experiments. All mice were housed in cages under specific pathogen-free conditions, and given water and standard laboratory chow ad libitum during the experiments. All animal handing procedures were in accordance with National Taiwan Ocean University (NTOU) guidelines. All procedures were approved by the Animal Care and Use Committee of NTOU.

### 4.2. Peptides, Reagents, and Antibodies

Reagents and chemicals were purchased from Sigma. Standard laboratory powders of methicillin (Cat. No. 51454; Sigma, St. Louis, MO, USA) and vancomycin (Cat. No. v2002; Sigma, St. Louis, MO, USA) were used and prepared according to the guidelines of the CLSI. Tilapia piscidin 4 (H-FIHHIIGGLFSAGKAIHRLIRRRRR-OH) was synthesized by GL Biochem (Shanghai, China) using a solid-phase procedure of Fmoc chemistry. Crude peptides were extracted, lyophilized, and purified by reverse-phase high performance liquid chromatography (HPLC). The molecular masses and purities of the purified peptides were respectively verified by mass spectroscopy and HPLC. Synthetic peptides at >95% purity were reconstituted in phosphate-buffered saline (PBS; pH 7.4) for the experiments. ELISA kits for Interleukin-1 (Cat. No. 559603, BD Biosciences, San Jose, CA, USA), tumor necrosis factor (Cat. No. 560478, BD Biosciences, San Jose, CA, USA), and Interleukin-6 (Cat. No. 555240, BD Biosciences, San Jose, CA, USA) were used to determine cytokine levels. Antibodies against VEGF (Cat. No. 550549, BD Biosciences, San Jose, CA, USA) were used for immunohistochemistry (IHC).

### 4.3. Cell Proliferation

Cells were cultured at a density of 5 × 10^4^ cells per well in flat-bottomed 96-well plates, and supplemented with various combinations of TP4. After 24 h, cell viability were measured with neutral red (Cat. No. N4638; Sigma, St. Louis, MO, USA) uptake assay, Cytotoxicity Detection Kit (LDH) (Cat. No. 11644793001; Roche Applied Science, Indianapolis, IN, USA), and CellTiter 96 Aqueous One Solution (Cat. No. G1111; Promega, Madison, WI, USA), following the vendors’ instructions.

### 4.4. Measurement of Collagen I, Collagen III, KGF, Keratin 10, and Keratin 17 Expression

Total RNA was isolated from HaCaT and Hs-68 cells, and purified using a Qiagen RNeasy kit. Reverse transcription into cDNA was performed with an iScript cDNA Synthesis Kit (Cat. No. 1708991; BIO-RAD, Hercules, CA, USA) according to the manufacturer’s recommendations. Real-time polymerase chain reaction (PCR) was performed to analyze gene expression, using 0.5 mL of cDNA, 2× iQSYBR^®^ Green Supermix (Cat. No. 1708882; BIO-RAD, Hercules, CA, USA), and 500 nM of forward and reverse primers against selected genes or GAPDH (reference gene), according to the instructions of the manufacturer. Quantitative PCR was performed under the following conditions: 40 cycles of 1 min at 95 °C, 30 s at 55 °C, and 1 min at 72 °C. The threshold cycle number (Ct) was calculated with BIO-RAD software. Relative transcript quantities were calculated using the ΔCt method with GAPDH as the internal reference gene. ΔCt is the difference in the threshold cycles of messenger (m) RNA for selected genes relative to those of GAPDH mRNA. Real-time PCR was performed in triplicate for each experimental group.

Primer sequences:
GAPDHSenseCGCTCTCTGCTCCTCCTGTTC(Nm_002046)AntisenseTTGACTCCGACCTTCACCTTCCCollagen ISenseACAGGGCGACAGAGGCATAAAG(NM_000088)AntisenseCCAGGAGCACCAGCAGAGCCollagen IIISenseTCCAAAGGGTGACAAGGGTGAAC(NM_000090)AntisenseAGGAGGACCAATAGGACCAGTAGGKGFSenseGCAACTGAACTTACTACGAACTG(S81661)AntisenseTCATTGACCTCTTCCTATCTGTGKeratin 10SenseCTGCGTAGGGTGCTGGATGAG(AF264085)AntisenseTTCCTCCTCGTGGTTCTTCTTCAGKeratin 17SenseCTGGCTGCTGATGACTTCC(NM_000422)AntisenseCCTCCTCGTGGTTCTTCTTC

### 4.5. Tilapia Piscidin 4 Peptides and Bacteriostatic Analysis

Minimum inhibitory concentration (MIC) antimicrobial assays were performed using standard protocols [33]. For MIC assessment, compounds were diluted to final concentrations of 100, 50, 25, 12.5, 6.25, 3.125, 1.582, or 0.78 μg/mL. Twenty microliters of each dilution were mixed in a microtiter plate well with 20 μL of the appropriate bacterial indicator suspension, and 160 μL of Trypticase Soy Broth (TSB) for MRSA, to a total volume of 200 μL. Three replicates were examined for MRSA, compound, and concentration. Positive controls contained water instead of compounds, and negative controls contained compounds without bacterial suspensions. Microbial growth was automatically determined by optical density measurement at 600 nm (Bioscreen C; Labsystem, Helsinki, Finland). Microplates (Cat. No. 3599; Corning, NY, USA) were incubated at 37 °C. Absorbance readings were taken at hourly intervals over a 24-h period, and the plates were shaken for 20 s before each measurement. The lowest compound concentration that resulted in zero growth by the end of the experiment was taken as the MIC.

### 4.6. In Vivo Toxicity

To determine the toxicity of TP4, TP4 was dissolved in phosphate-buffered saline (PBS; pH 7.4) and administered as intramuscular bolus injections in the left thigh (2 mg/mouse). Each group contained 10 mice. Controls were treated with PBS (control). Blood samples (0.2 mL) were collected on days 1, 3, and 6 after the final injection of TP4, and used to determine the serum levels of glutamic oxaloacetic transaminase (GOT), glutamic pyruvic transaminase (GPT), blood urea nitrogen (BUN), creatinine (CRE), total glucose (GLU), and creatine phosphokinase (CPK).

### 4.7. Therapeutic Use in a Mouse Model of MRSA Sepsis

Female Balb/c mice (6–8 weeks old) were injected intraperitoneally with 10^6^ CFU MRSA per mouse. Ten minutes after MRSA injection, mice were injected intraperitoneally with vancomycin (0.01 mg/g mouse body weight), methicillin (0.01 mg/g mouse body weight), or TP4 (0.005 mg/g mouse body weight). In a second set of experiments, mice were given intraperitoneal injections of TP4 (0.005 mg/g mouse body weight) at 10, 60, 120, or 180 min after MRSA injection. Survival rate and status were recorded every 24 h for up to 192 h. To examine bacterial dissemination, mice were sacrificed at 48 h after injection with antibiotics or TP4, and the bacterial numbers in blood, peritoneum, spleen, liver, and mesenteric lymph nodes were recorded. Colony counts from the diluted bacterial solutions were expressed relative to those at the start of treatment. These experiments consisted of four groups, and each group contained 10 mice.

### 4.8. Mouse Models of Wound Healing

Female Balb/c mice (6–8 weeks old) were used for wound healing experiments. All mice were housed individually to prevent fighting and further damage to the wounds, and they were provided with food and water *ad libitum*. Mice were maintained on a 12 h light: dark cycle at room temperature, and acclimatized to the environment for at least a week before use in experiments. All researchers wore caps, sterile gloves, gowns, and shoe covers when handling mice. Hair was removed from the back of the mice by shaving, and a full thickness wound (1 cm in diameter) was then created in the exposed region. Each wound was inoculated with 50 µL of broth mix containing 10^6^ CFU (colony forming units) of MRSA. At 5 min after inoculation, 50 µL TP4 (2 mg/mL) in a total volume of 0.1 mL were applied to the wound. Thirty minutes after treatment, wounds were covered with Tegaderm (3 M, St. Paul, MN, USA) to maintain uniformity, and to prevent the mice from removing the treatments. Based on initial experiments, we examined the wounds at 3, 7, 14, and 21 days post-injury, so as not to disturb the infection [27]. Such examinations captured the transitions from inflammatory to regenerative, and regenerative to resolving phases of wound healing [29]. Animals were subsequently euthanized by CO_2_ inhalation and the wounds assessed. Four individuals in each group were examined at each time point for each experiment. Each wound was measured and then removed from the animal, with unwounded skin taken from the contralateral dorsum as a control. Each biopsy was divided into six sections, with three sections being used for tensiometry and histology, and three sections for quantitative determination of microbial load. Wound healing studies were repeated in triplicate.

### 4.9. Wound Closure Measurements and Cell Proliferation Gene Expression

Tracings were taken immediately after injury. For uncontaminated wounds, wound size was determined every second day. For contaminated wounds, mice were euthanized at days 3, 5, or 19, and tracings of the wound edges were made. Wound areas were determined using the Macintosh Adobe Photoshop program, Histogram Analysis. The percentage of wound contraction was calculated as follows: % Wound contraction = (A_0_ − A_t_)/A_0_× 100, where A_0_ is the original wound area, and A_t_ is the area of wound at the time of biopsy, accordingly [30]. Cell proliferation gene expression was performed by real-time PCR, using the methods described in Section 4.4 above.

Primer sequences: GAPDHSenseCTCCAAGGAGTAAGAAACCC(GU214026.1)AntisenseTGGAAATTGTGAGGGAGATGEGFSenseCATATGTGATGGCTACTGCT(AF125256.1)AntisenseTTAATGTTCCTCAGGGAAGCTGF-βSenseCGTGCTCTTCTTCGACAATA(M57639)AntisenseAACATGAACAAACAGTCCCTVEGFSenseACCTTTGGGAAGAAGATGTC(AB086118.1)AntisenseCAATAGAACCCTCGAGTGAG

### 4.10. IHC and ELISA of Cytokines

Skin tissues were removed and fixed as previously described [34]. In brief, the cryosections were fixed with 4% formaldehyde, and the tissue samples were stained with VEGF. IHC was analyzed by three independent investigators. Images were taken using a BX-51 microscope (Olympus, Japan). ELISA was performed as previously described [32].

### 4.11. Statistical Analysis

All experiments were performed in triplicate on three biological replicates. All data are present as mean ± SEM. We used two-tailed Student’s *t* tests to determine significance between two groups. We performed analyses of multiple groups by one-way or two-way ANOVA with Bonferroni posttest, using GraphPad Prism Version 5. For all statistical tests, we considered p values less than 0.05 to be statistically significant. Groups of 10 mice were used for each treatment, and each experiment was repeated three times.

## 5. Conclusions

The use of TP4 may complement the use of antibiotics. Critically, TP4 is unlikely to induce resistance, is compatible with the use of antibiotics, and does not have any apparent immunotoxic effects. Moreover, TP4 induces proliferation of epithelial cells, which may be due to altered gene expression of collagen I, collagen III, keratinocyte growth factor (KGF), and keratin 10. In addition to its host defense function and modulatory effect on the innate immune system, TP4 may play an important role in reducing the risk of infection. Our model is valuable for future research on the pathophysiology of wound healing, as well as for testing new therapeutics for the treatment of bacterial infection during wound healing.

## References

[1] Theuretzbacher U., Toney J.H. (2006). Nature’s clarion call of antibacterial resistance: Are we listening?. Curr. Opin. Investig. Drugs.

[2] Spellberg B., Powers J.H., Brass E.P., Miller L.G., Edwards J.E. (2004). Trends in antimicrobial drug development: Implications for the future. Clin. Infect. Dis..

[3] Zasloff M. (2002). Antimicrobial peptides of multicellular organisms. Nature.

[4] Huang H.N., Pan C.Y., Rajanbabu V., Chan Y.L., Wu C.J., Chen J.Y. (2011). Modulation of immune responses by the antimicrobial peptide, epinecidinn(Epi)-1, and establishment of an Epi-1-based inactivated vaccine. Biomaterials.

[5] Peng K.C., Lee S.H., Hour A.L., Pan C.Y., Lee L.H., Chen J.Y. (2012). Five different piscidins from Nile tilapia, *Oreochromis niloticus*: Analysis of their expressions and biological functions. PLoS ONE.

[6] Acosta J., Carpio Y., Valdés I., Velázquez J., Zamora Y., Morales R., Morales A., Rodríguez E., Estrada M.P. (2014). Co-administration of tilapia alpha-helical antimicrobial peptides with subunit antigens boost immunogenicity in mice and tilapia (*Oreochromis niloticus*). Vaccine.

[7] O’Meara S., Cullum N., Majid M., Sheldon T. (2000). Systematic reviews of wound care management: (3) antimicrobial agents for chronic wounds; (4) diabetic foot ulceration. Health Technol. Assess..

[8] Pan C.Y., Wu J.L., Hui C.F., Lin C.H., Chen J.Y. (2011). Insights into the antibacterial and immunomodulatory functions of the antimicrobial peptide, epinecidin-1, against *Vibrio vulnificus* infection in zebrafish. Fish Shellf. Immunol..

[9] Huang T.C., Chen J.Y. (2013). Proteomic and functional analysis of zebrafish after administration of antimicrobial peptide epinecidin-1. Fish Shellf. Immunol..

[10] Singer A.J., Clark R.A.F. (1999). Cutaneous wound healing. N. Engl. J. Med..

[11] Michelson P.H., Tigue M., Panos R.J., Sporn P.H. (1999). Keratinocyte growth factor stimulates bronchial epithelial cell proliferation *in vitro* and *in vivo*. Am. J. Physiol. Lung Cell Mol. Physiol..

[12] Pawankar R. (2001). Epithelial cells as immunoregulators in allergic airway diseases. Curr. Opin. Allergy Clin. Immunol..

[13] Puddicombe S.M., Polosa R., Richter A., Krishna M.T., Howarth P.H., Holgate S.T., Davies D.E. (2000). Involvement of the epidermal growth factor receptor in epithelial repair in asthma. FASEB J..

[14] Aarbiou J., Verhoosel R.M., van Wetering S., de Boer W.I., van Krieken J.H., Litvinov S.V., Rabe K.F., Hiemstra P.S. (2002). Human neutrophil defensins induce lung epithelial cell proliferation *in vitro*. J. Leukoc. Biol..

[15] Aarbiou J., Verhoosel R.M., van Wetering S., de Boer W.I., van Krieken J.H., Litvinov S.V., Rabe K.F., Hiemstra P.S. (2004). Neutrophil defensins enhance lung epithelial wound closure and mucin gene expression *in vitro*. Am. J. Respir. Cell Mol. Biol..

[16] Houghton P.J., Hylands P.J., Mensah A.Y., Hensel A., Deters A.M. (2005). *In vitro* tests and ethnopharmacological investigations: Wound healing as an example. J. Ethnopharmacol..

[17] Martin A. (1996). The use of antioxidants in healing. Dermatol. Surg..

[18] Ruszczak Z. (2003). Effect of collagen matrices on dermal wound healing. Adv. Drug Deliv. Rev..

[19] McGowan K.M., Tong X., Colucci-Guyon E., Langa F., Babinet C., Coulombe P.A. (2002). Keratin 17 null mice exhibit age and strain-dependent alopecia. Genes Dev..

[20] Sauder D.N., Kilian P.L., McLane J.A., Quick T.W., Jakubovic H., Davis S.C., Eaglstein W.H., Mertz P.M. (1990). Interleukin-1 enhances epidermal wound healing. Lymphokine Res..

[21] Galko M.J., Krasnow M.A. (2004). Cellular and genetic analysis of wound healing in *Drosophila* larvae. PLoS Biol..

[22] Beanes S.R., Dang C., Soo C., Ting K. (2003). Skin repair and scar formation: The central role of TGF-beta. Expert Rev. Mol. Med..

[23] Herbst R.S. (2004). Review of epidermal growth factor receptor biology. Int. J. Radiat. Oncol. Biol. Phys..

[24] Broughton G., Janis J.E., Attinger C.E. (2006). Wound healing: An overview. Plast. Reconstr. Surg..

[25] Fox J.L. (2013). Antimicrobial peptides stage a comeback. Nat. Biotechnol..

[26] Yang D., Biragyn A., Hoover D.M., Lubkowski J., Oppenheim J.J. (2004). Multiple roles of antimicrobial defensins, cathelicidins, and eosinophil-derived neurotoxin in host defense. Annu. Rev. Immunol..

[27] Hebda P.A., Whaley D., Kim H.G., Wells A. (2003). Absence of inhibition of cutaneous wound healing in mice by oral doxycycline. Wound Repair Regen..

[28] Babu M., Wells A. (2001). Dermal-epidermal communication in wound healing. Wounds.

[29] Hsu J.C., Lin L.C., Tzen J.T., Chen J.Y. (2011). Pardaxin-induced apoptosis enhances antitumor activity in HeLa cells. Peptides.

[30] Kirker K.R., Luo Y., Nielson J.H., Shelby J., Prestwich G.D. (2002). Glycosaminoglycan hydrogel films as bio-interactive dressings for wound healing. Biomaterials.

[31] Bird T.A., Saklatvala J. (1989). IL-1 and TNF transmodulate epidermal growth factor receptors by a protein kinase C-independent mechanism. J. Immunol..

[32] Huang H.N., Rajanbabu V., Pan C.Y., Chan Y.L., Wu C.J., Chen J.Y. (2013). Use of the antimicrobial peptide Epinecidin-1 to protect against MRSA infection in mice with skin injuries. Biomaterials.

[33] Cao L., Dai C., Li Z., Fan Z., Song Y., Wu Y., Cao Z., Li W. (2012). Antibacterial activity and mechanism of a scorpion venom peptide derivative *in vitro* and *in vivo*. PLoS ONE.

[34] Yates C.C., Whaley D., Babu R., Zhang J., Krishna P., Beckman E., Pasculle A.W., Wells A. (2007). The effect of multifunctional polymer-based gels on wound healing in full thickness bacteria-contaminated mouse skin wound models. Biomaterials.

